# Editorial: Atrial Fibrillation: selection of management strategy and evaluation of outcomes -volume II

**DOI:** 10.3389/fcvm.2026.1859934

**Published:** 2026-07-14

**Authors:** Rui Providencia, Alexandre Almorad

**Affiliations:** 1 Institute of Health Informatics Research, University College London, London, United Kingdom; 2 St. Bartholomew’s Hospital, Barts Heart Centre, Barts Health NHS Trust, London, United Kingdom; 3 Department of Cardiology, Hospital da Luz Arrábida, Vila Nova de Gaia, Portugal; 4 Heart Rhythm Management Centre, Postgraduate Program in Cardiac Electrophysiology and Pacing, Universitair Ziekenhuis Brussel - Vrije Universiteit Brussel, European Reference Networks Guard-Heart, Brussels, Belgium; 5 Heart Rhythm Management Unit, Department of Cardiology, Centre Universitaire St Pierre, Université Libre de Bruxelles, Brussels, Belgium

**Keywords:** atrial fibrillation, comorbidities, epidemiology, left atrial appendage (LAA), obesity

**Figure 1:**
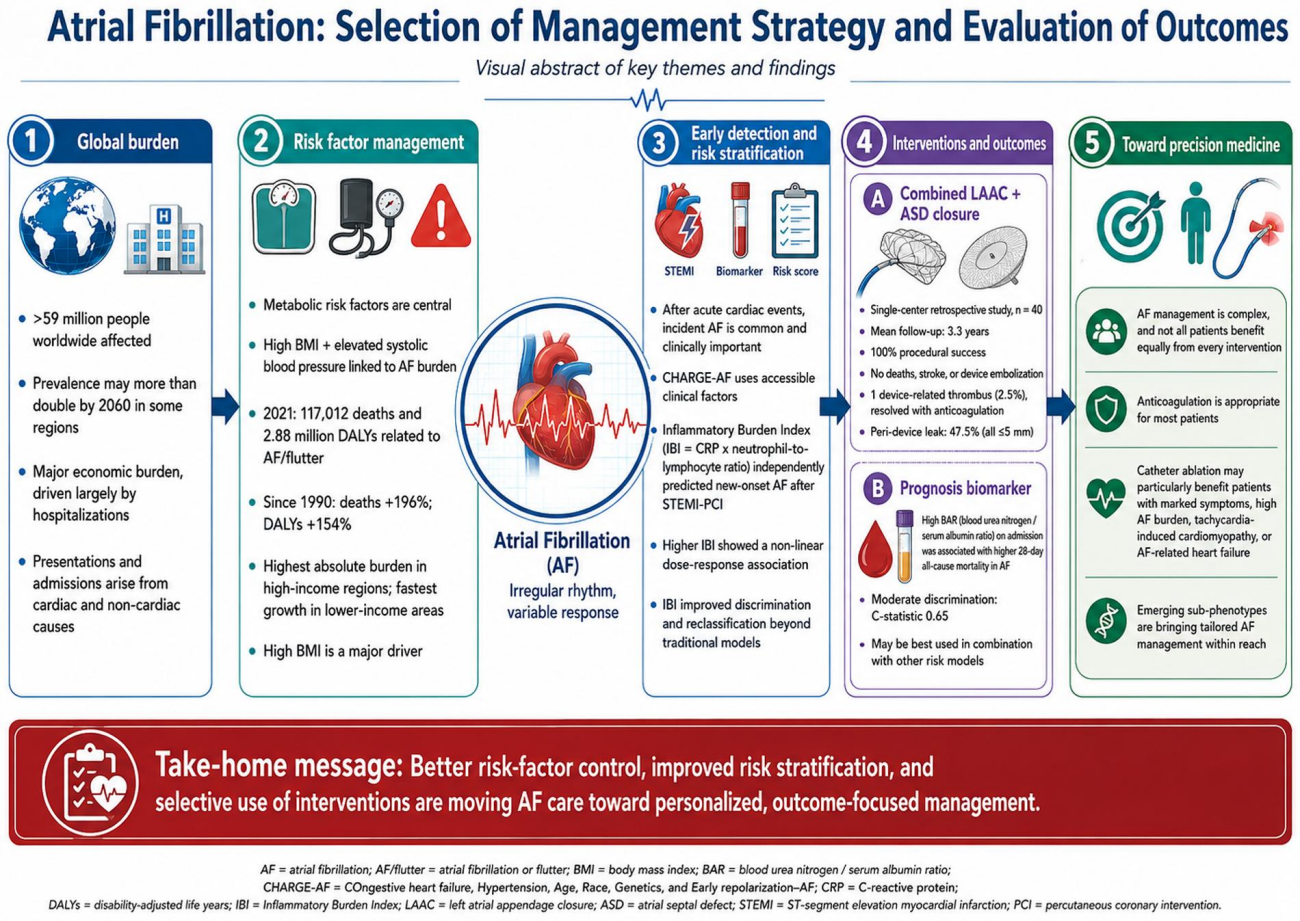
Visual summary of the editorial.

Atrial fibrillation (AF) is a rapidly growing global epidemic. According to the *Global Burden of Disease* (GBD) study, AF affects over 59.0 million individuals worldwide (1), with its prevalence in certain regions projected to more than double by 2060 (2). This condition imposes a significant economic burden on healthcare systems, primarily driven by hospitalizations (3). A unique feature of AF is that its clinical presentation, reasons for primary care visits, and causes of hospitalization stem from diverse cardiac and non-cardiac sources (4). Consequently, improving management strategies and rigorously assessing their impact on patient outcomes is essential. This second volume of the special Research Topic, “*Atrial Fibrillation: Selection of Management Strategy and Evaluation of Outcomes,”* aims to address these critical challenges (Figure 1).

Risk factor management is central to the treatment of AF. In recent years, obesity has garnered significant medical attention, largely due to the transformative impact of GLP-1 receptor agonists. An analysis of the GBD study published in this journal demonstrated that metabolic risk factors, particularly high body mass index (BMI) and elevated systolic blood pressure, accounted for 117,012 deaths and 2.88 million DALYs related to AF and flutter in 2021 (Wang et al.). This reflects a staggering increase of 196% and 154%, respectively, since 1990. While high-income regions still bear the highest absolute burden, the most rapid growth occurred in lower-income areas, with high BMI emerging as the primary driver of this trend.

Cardiac comorbidities and complications are highly prevalent in patients with or at risk for AF (5). Incident AF is common following acute cardiac events, such as myocardial infarction (4), and these individuals face a heightened risk of AF-related complications. It is therefore essential to identify patients at high risk of developing AF in these settings and to develop strategies for early detection. The CHARGE-AF score, which utilizes accessible clinical risk factors, is a prominent example of such a tool (6). However, there is still significant potential to enhance its discriminative power by incorporating complex biomarkers. In this issue, Liu et al. identified the Inflammatory Burden Index (IBI) as an independent risk factor for new-onset AF in a cohort of 696 patients with ST-elevation myocardial infarction (STEMI) undergoing percutaneous coronary intervention. Calculated by multiplying C-reactive protein levels by the neutrophil-to-lymphocyte ratio, the IBI integrates systemic inflammation with immune balance. Their study demonstrated a non-linear dose-response relationship between elevated IBI and new-onset AF, significantly improving risk stratification over traditional models, as evidenced by improvements in AUC, NRI, and IDI metrics.

Despite initial enthusiasm, recent trials have introduced some uncertainty regarding left atrial appendage closure (LAAC) in unselected AF populations (7–10). However, specific patient subsets may derive a more intuitive benefit. Wang et al., in a retrospective single-center study, demonstrated that a “one-stop” combined procedure for percutaneous LAAC and atrial septal defect (ASD) closure is both safe and feasible. Following forty patients over a mean follow-up of 3.3 years. The authors reported a 100% procedural success rate and absence of deaths, stroke or device embolization. One device-related thrombus (2.5%) that resolved after treatment with oral anticoagulants for an additional 3 months, and a high rate of peri-device leak (19 out of 40 cases; 47.5%; all ≤ 5 mm) was observed, which according to the authors may be explained by the implementation of CT surveillance protocols, which are deemed superior to transoesophageal echocardiogram in detecting subtle peri-device leaks.

Furthermore, incident AF is associated with an ominous prognosis, including higher mortality rates compared to the general population (11). Identifying at-risk patients remains a significant challenge. Huang et al. utilized MIMIC-IV data in a retrospective cohort study, which indicated that a high blood urea nitrogen-to-serum albumin ratio (BAR) upon admission was independently associated with an increased 28-day all-cause mortality risk in AF patients (12). The discriminative power of BAR was moderate (C-statistic 0.65), suggesting that its integration with other risk models may be necessary to optimize performance.

As it stands, AF management remains highly complex. Recent trial evidence suggests that interventions can significantly impact outcomes such as stroke and mortality (13–15); however, not all patients benefit equally from every intervention. While anticoagulants are suitable for most due to the rarity of absolute contraindications, patients with more pronounced symptoms, higher AF burden, and tachycardia-induced cardiomyopathy or AF-related heart failure may be some of the primary beneficiaries of catheter ablation. Although personalized therapy has long been an elusive goal, the knowledge acquired in recent years is gradually enabling the identification of AF sub-phenotypes. This progress brings us closer to the era of precision medicine, suggesting that tailored AF management is finally within reach.
